# Generative Artificial Intelligence in Medical Training: Utilization Patterns Across Knowledge, Patient Care, Systems Reasoning, and Innovation

**DOI:** 10.1007/s40670-025-02623-1

**Published:** 2026-01-04

**Authors:** Grace L. Park, Gary L. Beck Dallaghan, Joe Bradley, Qasim Sikander, Hannah Jung, Kevin Zhang, Gregory Polites, Janet Jokela

**Affiliations:** 1 Department of Biomedical and Translational Science, Carle Illinois College of Medicine/University of Illinois, Urbana, IL 61801 USA; 2 Department of Bioengineering, Grainger College of Engineering, Carle Illinois College of Medicine, University of Illinois, Urbana, IL USA; 3 Carle Illinois College of Medicine/University of Illinois, Urbana, IL USA; 4 Department of Clinical Sciences, Carle Illinois College of Medicine/University of Illinois, Urbana, IL USA

**Keywords:** Artificial intelligence, Generative AI, Medical education, Competencies, Innovation

## Abstract

Since 2022, generative artificial intelligence (AI) use has grown rapidly across many sectors, including medical education. While prior research has explored perceptions of AI, the understanding of AI use amongst medical trainees has been limited. This study surveyed medical trainees to better identify the patterns of generative AI use. Results showed varying patterns based on the phase of training, with ChatGPT emerging as the predominantly used platform across all phases. While awareness of AI policies was limited, the majority reported efforts for responsible use of AI. Implications include understanding of equitable access and onboarding regarding AI use policies.

## Introduction

The landscape of medical education is rapidly evolving with technological advancements, particularly through the integration of generative AI systems influencing how future healthcare professionals learn, study, and practice medicine. As healthcare information continues to proliferate at unprecedented rates, with global scientific research output growing by approximately 4% annually and reaching 3.3 million publications in 2022 [1] medical trainees are exploring various tools and resources to navigate this information landscape. The technology acceptance model which highlights the impact of perceived usefulness and ease of use can give a framework in understanding the patterns of adoption of these various tools[2]*.* The emergence of generative AI models (such as open source ChatGPT in November 2022) has introduced new options for medical education across all levels of training, from pre-clerkship students to trainees in residency programs [3]. Though there have been several studies assessing medical students’ perceptions of AI [4–8] at the time of our study, there was limited understanding of the utilization patterns of generative AI amongst medical trainees.

## Background

This study examines generative AI utilization patterns among medical trainees in different phases: undergraduate preclinical, undergraduate clinical, and graduate medical education. The study analyzes how trainees at each phase incorporate generative AI tools into their educational activities, study habits, patient care approaches, and research endeavors. The research questions guiding this study were: (1) How do medical trainees across different phases utilize generative AI? and (2) What type of generative AI platforms are they using? Understanding how these choices reflect learner priorities and preferences will provide insight into the evolving relationship between AI and medical education, informing future discussions about the role of generative AI in medical curricula.

## Methods

A cross-sectional study was conducted among medical trainees at Carle Illinois College of Medicine (CIMED) and Carle Health in Champaign-Urbana, Illinois from February to May 2025. Recruitment was done through distribution of flyers with QR codes in the student and resident lounges as well as direct distribution through class listservs. Inclusion criteria for recruitment were to be 18 years and older and a medical student or resident at Carle Illinois College of Medicine or Carle Health. 

Though there have been several surveys conducted to assess perceptions of AI in medical education [4–6], we were unable to identify a survey addressing trainee utilization patterns of AI in medical education at the time this study was conceived. This led to the development of an 18-item novel survey with the input from content experts in medical education and educational survey design. The survey was semi-structured with sections exploring utilization patterns in three different domains aligned with the Accreditation Council for Graduate Medical Education and the Association of American Medical Colleges core competencies: medical knowledge, patient care, health systems reasoning or systems-based practice [9, 10]. As CIMED is an institution that has the world’s first engineering integrated medical curriculum with the mission to train physician innovators [11], we also added the domain of research and innovation. The survey consisted of questions regarding familiarity with policies regarding generative AI, types of platforms utilized, and contexts in which generative AI are used as well as a question for each domain capturing in what capacities generative AI was being used and how it ranked in comparison to other more traditional educational sources. Questions regarding frequency of practices such as checking output data, verifying input data and sources, and evaluating for bias in output data were asked as a 5-point Likert scale (1 = Never to 5 = Always).

The survey was administered using Qualtrics (Qualtrics, 2025). The survey was open for 3 months, with a reminder email sent one time. Participation was voluntary and the survey was anonymous. The University of Illinois Urbana-Champaign Institutional Review Board reviewed and approved of this study (IRB No.250089).

Survey data were analyzed using a combination of descriptive and inferential statistics. Demographic data was summarized along with other questions. The ranked items were analyzed using Kruskal–Wallis H test to determine if there were differences in how pre-clinical students, clinical students, and residents rated their use of generative AI in different contexts. The analyses were conducted using IBM SPSS v29 (IBM Corp, 2022).

## Results

Among 347 eligible trainees, there was a total of 49 respondents to the survey. Out of the 49, 36 were medical students and 13 were resident physicians. Most of the survey respondents were in the 20–29 age range (76%), followed by ages 30–39 (18%). Most students in all stages of training indicated being unaware or unsure of AI policies at their medical school (92%) or at the health system where they practice (94%).

Student respondents who use generative AI indicated using ChatGPT (*N* = 42, 85.7%), OpenAI’s large language model. Open Evidence, an AI platform made for physicians at the point-of-care, was also popular (*N* = 19, 38.8%). Google’s Gemini, which is easily accessible through a Google search or within written documents, including Google Docs or Gmail, was next (*N* = 18, 36.7%).

**Fig. 1:**
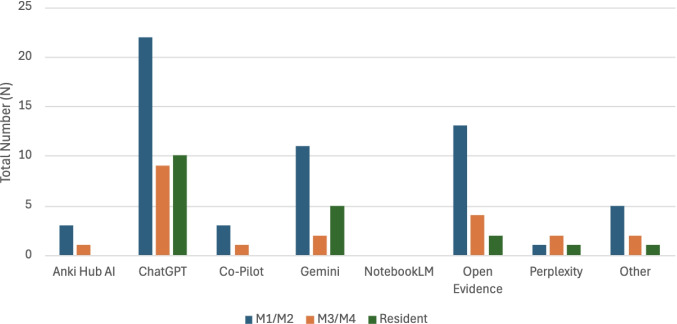
Utilization of generative AI platforms by training level

Interestingly, a less popular platform was Anki Hub AI (*N* = 4, 8.16%), despite the vast exposure to Anki that medical students receive. Looking at the breakdown based on levels of training, Fig. 1 shows that no resident physicians indicated using Anki Hub AI, perhaps because they do not rely on Anki to learn information as much as medical students do.

Other generative AI platforms that students use include Claude, DeepNote, GitHub Copilot, DeepSeek, and Grok.

**Fig. 2:**
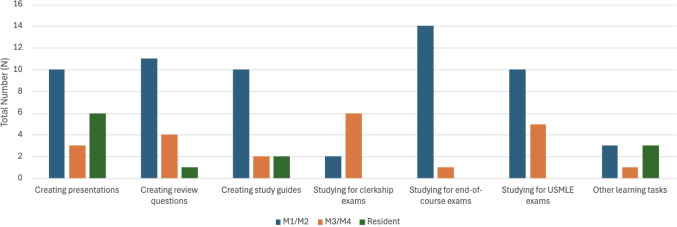
Utilization of generative AI for daily learning tasks by training level

All the respondents who used generative AI reported that they use it for daily learning tasks related to medical knowledge. As shown in Fig. 2, the use of AI was evenly distributed from creating presentations (20%), creating review questions (17%), studying for end-of-course exams (16%), creating study guides (16%), studying for USMLE exams (16%), and studying for clerkship exams (8%). Many M1s, M2s (*N* = 10), and most residents (*N* = 6) used generative AI to create presentations. M1s and M2s also use generative AI to write review questions (*N* = 11) or study guides (*N* = 10), and primarily to answer questions while studying for end-of-course (*N* = 14) or USMLE exams (*N* = 10). M3s and M4s also use generative AI for the same tasks, but they primarily use it to study for clerkship exams (*N* = 6).

Most survey participants (74%) used generative AI for daily patient care tasks, a close-even distribution of determining a diagnosis (19%), a management plan (18%), checking for drug-drug interactions (15%), advice for responding to situations (14%), and addressing SDoH (6%).

A little more than half of the survey participants (52%) reported using generative AI for health system reasoning, from finding resources for patients (23%), navigating health insurance (15%), finding costs for procedures or tests (8%), and finding costs for medications (6%).

A large majority of study participants used generative AI for research and innovation (88%), broken down into brainstorming (37%), development of ideas (26%), and data analysis (21%).

## Comparison of Ranked Items

A series of Kruskal–Wallis H tests were conducted to examine differences in the perceived usefulness of AI and the use of AI in comparison to other resources rankings among three independent groups: pre-clinical students, clinical students, and residents. See Table 1 for more details.

**Table 1:** Comparison of ranked items

	Median rank				
	M1/M2	M3/M4	Resident	H(2)	Sig
Contexts Al most useful					
Medical Knowledge	1	1	1	2.55	0.279
Patient Care	4	3/4	3	9.48	0.009*
Research & Innovation	2	2	2/3	1.54	0.463
Understanding Health Systems	3	3	4	0.25	0.884
Resource to study medical knowledge					
Generative Al	3/4	2/3	4	0.495	0.781
School lectures	2	4	3	15.43	< 0.001*
Textbooks or other assigned reading	4	2	1/2	10.22	0.006*
Third-party resources	1	1	3	12.23	0.002*
Resources to guide patient care decisions					
Clinical databases	1	1	1	3.35	0.187
Faculty or attendings	13	2/3	2	5.73	0.057
Generative Al	5	5	5	2.83	0.243
Other healthcare team members	3	4	4	2.62	0.269
Resources to understand health systems					
Faculty or attendings	1	3	2	3.00	0.223
Generative Al	6	6	5/6	3.54	0.170
Google or other search engines	2	4	1	3.19	0.203
Other healthcare team members	3	1/2	1/3/4/5	1.30	0.523
Pricing websites or apps	5	1/2/5	6	3.32	0.191
Residents	4	2/4/6	4	0.09	0.956
Resources for research and innovation					
Faculty or attendings	3	3	2	0.53	0.766
Generative Al	2/4	2/4	3	0.57	0.754
Google or other search engines	1	1	1	1.67	0.433
Residents	4	4	4	0.10	0.950

*Statistically significant differences (*p* <.05). “Not using AI” was omitted from the comparison. Ranking was done as 1 being highest priority/most used for use and the final number is the lowest priority/least used

Participants ranked the usefulness of AI across five contexts: medical knowledge, patient care, research and innovation, and understanding health systems. A statistically significant difference was found for patient care (H(2) = 9.48, *p* = 0.009), with residents more frequently ranking it as their top priority compared to the other groups.

Significant ranking differences were found in the use of school lectures (H(2) = 15.43, *p* < 0.001), textbooks or assigned readings (H(2) = 10.22, *p* = 0.006), and third-party resources (H(2) = 12.23, *p *= 0.002). Pre-clinical students ranked these resources higher than clinical students and residents. No significant difference was found for generative AI (H(2) = 0.495, *p* = 0.781).

When ranking resources guiding patient care decisions, participants could rank the following: faculty or attendings, residents, clinical databases, generative AI, or other healthcare team members. No statistically significant differences were found among the groups for any of the resources evaluated.

Similarly, when ranking resources for understanding health systems (faculty or attendings, generative AI, search engines, other healthcare team members, pricing websites/apps, or residents) as well as for research and innovation (faculty or attendings, generative AI, search engines, or residents), there were no statistically significant differences among the rankings.

## Using Generative AI Responsibly

One of the first disclaimers users see when prompting generative AI is that it makes mistakes. It is critical to avoid such mistakes with patients’ lives at stake, especially when there are texts, journals, and databases available with reliable and current information. Thus, we asked medical students and resident physicians who use generative AI whether they 1) cross-reference the output by AI with validated textbooks or sources, 2) evaluate the AI’s output for bias, 3) verify initial input data, and 4) verify sources and references that the AI provides. 67% of respondents indicated that they cross-reference the output by AI with validated textbooks or sources most of the time or always. *60%* of residents indicated that they evaluate the AI’s output for bias most of the time or always, compared to 45% of medical students. This discrepancy may come from medical students utilizing generative AI more for obtaining straightforward medical knowledge rather than complex questions related to patient communication or management where issues like age-or race-related bias may be more apparent. 72% percent of M3s, M4s, and residents indicated that they verify their initial input data most of the time or always. M1s and M2s were split equally on never or sometimes verifying their initial input data vs. most of the time or always. This may point to M3s, M4s, and residents understanding that they are using generative AI in more critical situations and should ensure the information they are inputting is correct. 77% of respondents indicated that they verify sources and references that AI provides most of the time or always.

## Discussion

The utilization of generative AI in medical education varies by training phase and specific educational context. Applying the technology acceptance model's constructs of perceived usefulness and perceived ease of use reveals consistent patterns, indicating that trainees' adoption of generative AI is closely influenced by their immediate context, such as classroom-based learning versus patient care activities. In the pre-clerkship years, generative AI use is often focused on the domain of medical knowledge to create study guides, review questions, and presentations, and assist with understanding complex concepts. During clinical clerkships and residency, utilization of generative AI shifts toward patient care and systems-based practice with trainees using it to support clinical reasoning, diagnostic skills, and patient management. For all levels of training, generative AI applications can help with literature searches and brainstorming for research. The generative AI platforms used vary with a predominance toward ChatGPT. It is not clear from our study whether learners are using the free or subscription versions of these generative AI platforms. This distinction is important, as subscription-based versions often offer enhanced privacy settings compared to free versions, which may store or use user input for model training. The access to subscription-based platforms may be limited by financial or institutional support, potentially creating disparities in the ability for learners to utilize these generative AI tools [12, 13]. With the ever-increasing number of platforms being developed, it will be important to continue to reassess this question. Overall, learners approach generative AI with a degree of caution, particularly in relation to its accuracy. This caution appears to be more pronounced among residents, who may be more aware of the potential clinical implications of relying on inaccurate information. This pattern aligns with recent literature indicating that, in the high-risk context of medicine, trust and credibility are central to the adoption of AI, measured by the constructs of perceived risk and AI trust [14]*.*

Future directions should include a continued examination of the patterns of utilization of generative AI across different core competency domains as this has direct implications for curriculum development and support. In the development of medical knowledge, ensuring equitable access to generative AI tools for students is crucial to support learning across diverse educational settings. For the competency of patient care, the increasing use of generative AI by trainees highlights the need for education on its limitations, including potential biases and the risk of misinformation. Additionally, as generative AI becomes more integrated into healthcare systems, preparing learners to navigate evolving technologies and institutional policies is vital for fostering competency in systems-based practice. Given the rapid pace of AI utilization, development and education regarding policies related to AI as well as structured onboarding at the institutional level will be essential to ensure responsible and ethical use of these tools.

### Limitations

This study has several limitations. First, it was conducted at a single institution, which may limit the generalizability of the finding,. Second, the number of participants was not evenly distributed across the different years of training, which may have influenced the results. Third, participation in the study was anonymous and voluntary, introducing the potential for selection bias as well as limited respondents. Limited responses and uneven representation may have been from the method of distribution of the survey which was not directly sent to each eligible trainee and thus inability to verify whether all eligible individuals were reached by the recruitment efforts.

## Data Availability

The survey instrument and de-identified survey response data used in this study are available from the corresponding author on reasonable request, subject to institutional and ethical approval.
